# Explainable Artificial Intelligence for Neuroscience: Behavioral Neurostimulation

**DOI:** 10.3389/fnins.2019.01346

**Published:** 2019-12-13

**Authors:** Jean-Marc Fellous, Guillermo Sapiro, Andrew Rossi, Helen Mayberg, Michele Ferrante

**Affiliations:** ^1^Theoretical and Computational Neuroscience Program, National Institute of Mental Health, National Institutes of Health, Bethesda, MD, United States; ^2^Department of Psychology and Biomedical Engineering, University of Arizona, Tucson, AZ, United States; ^3^Department of Electrical and Computer Engineering, Duke University, Durham, NC, United States; ^4^Executive Functions and Reward Systems Program, National Institute of Mental Health, National Institutes of Health, Bethesda, MD, United States; ^5^Center for Advanced Circuit Therapeutics, Icahn School of Medicine at Mount Sinai, New York, NY, United States; ^6^Computational Psychiatry Program, National Institute of Mental Health, National Institutes of Health, Bethesda, MD, United States

**Keywords:** explain AI, closed-loop neurostimulation, computational psychiatry, behavioral paradigms, machine learning, neuro-behavioral decisions systems, data-driven discoveries of brain circuit theories

## Abstract

The use of Artificial Intelligence and machine learning in basic research and clinical neuroscience is increasing. AI methods enable the interpretation of large multimodal datasets that can provide unbiased insights into the fundamental principles of brain function, potentially paving the way for earlier and more accurate detection of brain disorders and better informed intervention protocols. Despite AI’s ability to create accurate predictions and classifications, in most cases it lacks the ability to provide a mechanistic understanding of how inputs and outputs relate to each other. Explainable Artificial Intelligence (XAI) is a new set of techniques that attempts to provide such an understanding, here we report on some of these practical approaches. We discuss the potential value of XAI to the field of neurostimulation for both basic scientific inquiry and therapeutic purposes, as well as, outstanding questions and obstacles to the success of the XAI approach.

## Introduction

One of the greatest challenges to effective brain-based therapies is our inability to monitor and modulate neural activity in real time. Moving beyond the relatively simple open-loop neurostimulation devices that are currently the standard in clinical practice (e.g., epilepsy) requires a closed-loop approach in which the therapeutic application of neurostimulation is determined by characterizing the moment-to-moment state of the brain (Herron et al., 2017). However, there remain major obstacles to progress for such a closed-loop approach. For one, we do not know how to objectively characterize mental states or even detect pathological activity associated with most psychiatric disorders. Second, we do not know the most effective way to improve maladaptive behaviors by means of neurostimulation. The solutions to these problems require innovative experimental frameworks leveraging intelligent computational approaches able to sense, interpret, and modulate large amount of data from behaviorally relevant neural circuits at the speed of thoughts. New approaches such as computational psychiatry (Redish and Gordon, 2016; Ferrante et al., 2019) or ML are emerging. However, current ML approaches that are applied to neural data typically do not provide an understanding of the underlying neural processes or how they contributed to the outcome (i.e., prediction or classifier). For example, significant progress has been made using ML to effectively classify EEG patterns, but the understanding of brain function and mechanisms derived from such approaches still remain relatively limited (Craik et al., 2019). Such an understanding, be it correlational or causal, is key to improving ML methods and to suggesting new therapeutic targets or protocols using different techniques. Explainable Artificial Intelligence (XAI) is a relatively new set of techniques that combines sophisticated AI and ML algorithms with effective explanatory techniques to develop explainable solutions that have proven useful in many domain areas (Core et al., 2006; Samek et al., 2017; Yang and Shafto, 2017; Adadi and Berrada, 2018; Choo and Liu, 2018; Dosilovic et al., 2018; Holzinger et al., 2018; Fernandez et al., 2019; Miller, 2019). Recent work has suggested that XAI may be a promising avenue to guide basic neural circuit manipulations and clinical interventions (Holzinger et al., 2017b; Vu et al., 2018; Langlotz et al., 2019). We will develop this idea further here.

Explainable Artificial Intelligence for neurostimulation in mental health can be seen as an extension in the design of BMI. BMI are generally understood as combinations of hardware and software systems designed to rapidly transfer information between one or more brain area and an external device (Wolpaw et al., 2002; Hatsopoulos and Donoghue, 2009; Nicolelis and Lebedev, 2009; Andersen et al., 2010; Mirabella and Lebedev, 2017). While there is a long history of research in the decoding, analyses and production of neural signal in non-human primates and rodents, a lot of progress has recently been made to develop these techniques for the human brain both invasively and non-invasively, unidirectionally or bi-directionally (Craik et al., 2019; Martini et al., 2019; Rao, 2019). Motor decision making for example, has been shown to involve a network of brain areas, before and during movement execution (Mirabella, 2014; Hampshire and Sharp, 2015), so that BMI intervention can inhibit movement up to 200 ms after its initiation (Schultze-Kraft et al., 2016; Mirabella and Lebedev, 2017). The advantage of this type of motor-decision BMI is that it is not bound to elementary motor commands (e.g., turn the wheel of a car), but rather to the high-level decision to initiate and complete a movement. That decision can potentially be affected by environmental factors (e.g., AI vision system detecting cars on the neighboring lane) and internal state (e.g., AI system assessing the state of fatigue of the driver). The current consensus is that response inhibition is an emergent property of a network of discrete brain areas that include the right inferior frontal gyrus and that leverage basic wide-spread elementary neural circuits such a local-lateral-inhibition (Hampshire and Sharp, 2015; Mirabella and Lebedev, 2017). This gyrus, as with many other cortical structures, is dynamically recruited so that individual neurons may code for drastically different aspects of the behavior, depending of the task at hand. Consequently, designing a BMI targeting such an area requires the ability for the system to rapidly switch its decoding and stimulation paradigms as a function of environmental or internal state information. Such online adaptability needs of course to be learned and personalized to each individual patient, a task that is ideally suited for AI/ML approaches. In the sensory domain, some have shown that BMI can be used to generate actionable entirely artificial tactile sensations to trigger complex motor decisions (O’Doherty et al., 2012; Klaes et al., 2014; Flesher et al., 2017). Most of the BMI research work has, however, focused on the sensory motor system because of the relatively focused and well-defined nature of the neural circuits. Consequently, most of the clinical applications are focused on neurological disorders. Interestingly, new generations of BMIs are emerging that are focused on more cognitive functions such as detecting and manipulating reward expectations using reinforcement learning paradigms (Mahmoudi and Sanchez, 2011; Marsh et al., 2015; Ramkumar et al., 2016), memory enhancement (Deadwyler et al., 2017) or collective problem solving using multi-brain interfacing in rats (Pais-Vieira et al., 2015) or humans (Jiang et al., 2019). All these applications can potentially benefit from the adaptive properties of AI/ML algorithms and, as mentioned, explainable AI approaches have the promise of yielding basic mechanistic insights about the neural systems being targeted. However, the use of these approaches in the context of psychiatric or neurodevelopmental disorders has not been realized though their potential is clear.

In computational neuroscience and computational psychiatry there is a contrast between theory-driven (e.g., reinforcement learning, biophysically inspired network models) and data-driven models (e.g., deep-learning or ensemble methods). While the former models are highly explainable in terms of biological mechanisms, the latter are high performing in terms of predictive accuracy. In general, high performing methods tend to be the least explainable, while explainable methods tend to be the least accurate. Mathematically, the relationship between the two is still not fully formalized or understood. These are the type of issues that occupy the ML community beyond neuroscience and neurostimulation. XAI models in neuroscience might be created by combining theory- and data-driven models. This combination could be achieved by associating explanatory semantic information with features of the model; by using simpler models that are easier to explain; by using richer models that contain more explanatory content; or by building approximate models, solely for the purpose of explanation.

Current efforts in this area include: (1) identify how explainable learning solutions can be applied to neuroscience and neuropsychiatric datasets for neurostimulation, (2) foster the development of a community of scholars working in the field of explainable learning applied to basic neuroscience and clinical neuropsychiatry, and (3) stimulate an open exchange of data and theories between investigators in this nascent field. To frame the scope of this article, we lay out some of the major key open questions in fundamental and clinical neuroscience research that can potentially be addressed by a combination of XAI and neurostimulation approaches. To stimulate the development of XAI approaches the National Institute of Mental Health (NIMH) has released a funding opportunity to apply XAI approaches for decoding and modulating neural circuit activity linked to behavior^1^.

## Intelligent Decoding and Modulation of Behaviorally Activated Brain Circuits

**FIGURE 1:**
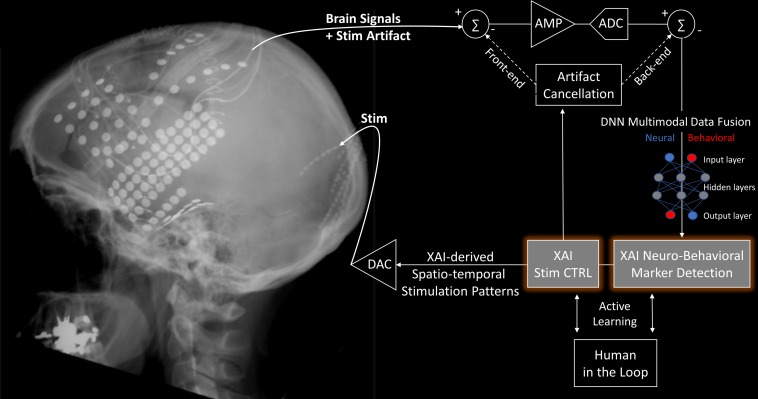
An XAI-enabled closed-loop neurostimulation process can be described in four phases: (1) System-level recording of brain signals (e.g., spikes, LFPs, ECoG, EEG, neuromodulators, optical voltage/calcium indicators), (2) Multimodal fusion of neural data and dense behavioral/cognitive assessment measures. (3) XAI algorithm using unbiasedly discovered biomarkers to provide mechanistic explanations on how to improve behavioral/cognitive performance and reject stimulation artifacts. (4) Complex XAI-derived spatio-temporal brain stimulation patterns (e.g., TMS, ECT, DBS, ECoG, VNS, TDCS, ultrasound, optogenetics) that will validate the model and affect subsequent recordings. ADC, Analog to Digital Converter; AMP, Amplifier; CTRL, Control; DAC, Digital to Analog Converter; DNN, Deep Neural Network. XRay picture courtesy Ned T. Sahin. Diagram modified from Zhou et al. (2018).

A variety of perspectives for how ML and, more generally AI could contribute to closed-loop brain circuit interventions are worth investigating (Rao, 2019). From a purely signal processing stand point, an XAI system can be an active stimulation artifact rejection component (Zhou et al., 2018). In parallel, the XAI system should have the ability to discover – in a data-driven manner – neuro-behavioral markers of the computational process or condition under consideration. Remarkable efforts are currently underway to derive biomarkers for mental health, as is the case for example for depression (Waters and Mayberg, 2017). Once these biomarkers are detected, and the artifacts rejected, the XAI system can generate complex feedback stimulation patterns designed and monitored (human in-the loop) to improve behavioral or cognitive performance (Figure 1). XAI approaches have also the potential to address outstanding biological and theoretical questions in neuroscience, as well as to address clinical applications. They seem well-suited for extracting actionable information from highly complex neural systems, moving away from traditional correlational analyses and toward a causal understanding of network activity (Yang et al., 2018). However, even with XAI approaches, one should not assume that understanding the statistical causality of neural interactions is equivalent to understanding behavior; a highly sophisticated knowledge of neural activity and neural connectivity is not generally synonymous with understanding their role in causing behavior.

**Fundamental neuroscientific questions that XAI could address**

• What are the biological mechanisms of memory storage and retrieval?
• What is the neural code and how is information transmitted between brain areas?
• What is the relationship between patterns of activity and behavior?
• Are there emergent properties of networks which are necessary for behavior?
• What are the relevant temporal and spatial scales necessary for decoding and modulating a given behavior?
• How should models account for the relationship between neurostimulation and physiological response, especially when that transfer function changes over time?

**Potential applications of XAI in computational psychiatry**

• Real time, closed-loop stimulation for DBS: ML algorithms could be trained to identify electrophysiological patterns that correspond to pathological states and apply patterned DBS to achieve a normative state.
• Development of inexpensive computerized assessments for diagnosing or characterizing prodromal risk for neuropsychiatric conditions such as psychosis, depression, PTSD, ADHD or autism.
• Personalized medicine approach: XAI could provide automated identification of sub-clusters of patients through analysis of multimodal data (e.g., imaging, behavioral) to enable individualized interventions and forecasting.
• Identifying clinical diagnostic groups; discovering individualized biomarkers for treatment selection; tracking and quantifying treatment response over time.

**Requirements to apply XAI approaches to neural circuit modulation**

• Analytic modeling of behavior to define precision targets for ML.
• Statistically robust brain metrics to dimensionally differentiate along the normative-to-aberrant continuum of activity.
• Methods for discovering potential causal relationships between neurons and between neural activity and behavior using large data sets.
• The inclusion of both central and peripheral nervous system dynamics (e.g., Vagal nerve stimulation or closed loop control of visceral organ systems).
• Linking of analytical models: For example, classification/brain-decoding models (SVM/MVPA) to theoretically-driven, encoding models or biological multiscale modeling.
• Technology required to determine the level of resolution (e.g., number of neurons) associated with a specific behavior. Technology required to monitor populations of cells across several brain regions chronically and simultaneously, while decoding the relevant biomarkers and delivering a modifying signal in real time.

**Beyond closed-loop neuro-behavioral modulation, unanswered questions relevant to the theoretical and practical applications of XAI:**

• How much data is needed to build/train an accurate and generalizable model?
• Can we build robust models to predict cognition for every possible describable cognitive function? For each cognitive function, can we build an effective neurostimulation strategy? If such models behave as predicted, how do we test their combinatorial properties? How to include the known multidimensional aspects of complex neuropsychiatric disorders into these emerging models. Will combinatorial models follow single behavior models? Will such models predict behaviors reliably trans-diagnostically?
• How do downstream neurons (i.e., reader/decoding mechanisms) interpret patterns of activity?
• Is it even possible to stimulate the brain exogenously in a manner that mimics endogenous activity?
• How best to move away from neo-phrenology and how to incorporate in our computational models the notion that the brain is a dynamical system, with all the significant computational challenges that this notion implies?
• What are the ethical considerations related to AI-assisted closed-loop stimulation?
• What are the legal considerations (e.g., FDA approval, liability) related to considering a continuously evolving AI-assisted closed-loop system a ‘medical device’?

## Can Ai Solutions Be Explainable/Interpretable?

The field is split about the potential and need for AI to be explainable and/or interpretable (Holzinger et al., 2017a; Jones, 2018). Some view AI as a tool for solving a technical problem but not necessarily useful for answering a scientific question. Others think it may indeed be possible for AI actions to be interpreted and/or understood by humans, but it depends on the level of understanding being sought. Decoding techniques are typically used to test whether sampled neural data contains information that allow prediction of a dependent variable. For example, if a decoder is reducible to a set of independent contributions from the signals of individual cells, then it may be entirely possible to map the population signal back to descriptive statistics of the individual neurons (e.g., firing rate). In this case, the decoder is interpretable within our understanding of neurophysiology. On the other hand, a solution derived from a decoder may be abstract and not map onto our understanding of the neural system. For this more likely scenario, an iterative process for interpretability may be required to force ML methods to fit models with specific interpretations. This could conceivably be achieved by incorporating data visualization techniques and statistical tools that would allow neuroscientists to assess the validity of data characteristics that were used to solve the problem.

A related question is whether AI solutions can be explainable to the point of providing mechanistic insights into how the brain is accomplishing a particular function or a set of complex behaviors. Presently, there is a significant gap between the performance of explainable biophysical models for prediction and that of more opaque ANNs. Is it reasonable to expect that the synthetic algorithms and architecture that AI systems use be informative of the underlying biological process? Can we assume the decoder is using the same information as the biological network (downstream brain areas)? Perhaps the parsimonious AI process is not the same as the brain process. It may be that AI solutions are explainable (in abstraction) but inherently uninterpretable in the context of the underlying biology. Irrespective, explanations can at a minimum give insights and help improve the AI performance.

## What Are the Next Steps Toward a Breakthrough? What Are the Major Challenges?

Three major areas in need of advancement can be identified: the need for richer datasets, more sophisticated models and methods, and cultural changes to further encourage collaborative efforts across scientific disciplines.

One of the challenges to building a durable theory of neural computation is that the foundational empirical data are limited or incomplete and, in the case of neural data, often sub-sampled (spatially and temporally). There is a general need for large, high-dimensional data sets to create models with a high degree of predictability. For example, such datasets could include quantitative data from specific multimodal signals (e.g., neural activity, neurotransmitter release, receptor activation, immune, endocrine or behavioral responses) for long periods of time. Data acquisition should be expanded to capture the developmental trajectory of an organism and contextually relevant environmental factors (e.g., naturalistic settings). Technological advances in acquisition systems will be necessary for monitoring and modulating brain function continuously, over long timescales. In addition, an important next step is to achieve more accurate and higher resolution measures of behavioral states (e.g., perceptual, social, affective, motor). Improvements in data accessibility and ease of sharing will be critical for these efforts to succeed.

A second critical step to move the field forward is the advancement of models and methods. Currently, most models operate at a single level of analysis (e.g., cell biophysics). Multi-level modeling has been a notoriously hard task to achieve using classical methods (e.g., analytically linking biophysical models to neural mass models). To accurately represent the complexity of neural systems, there is a need for XAI models to bridge from cellular level mechanisms to behavior. To reach this goal, we need heuristics and methods for quantifying the performance of these models and tools that will help us understand the nature of input-output transformations between interacting brain networks. These could include new methods for unsupervised learning from multiple modalities of data, and both statistical and analytical methods for understanding the relationships discovered in these data at multiple levels of description. The potential of these models for both basic neuroscience and clinical applications will rely on the development of tools to improve their construct validity and interpretability.

Finally, there needs to be a cultural change in the scientific enterprise itself. There is a need for more opportunities that enable meaningful and enduring collaborations between neuroscientists, clinicians, theorist, and ML experts. Interdisciplinary collaborative efforts need to be recognized and supported by academic institutions and funding agencies (Vu et al., 2018). In addition, open sharing of data and code will be important for moving this field forward. Modelers, theoreticians, and data scientists need unfettered access to well-annotated datasets. It may also be useful to adopt industry approaches like crowdsourcing, “use case” proof-of-concept studies, and grand challenges to attract interest to this area of science and technology development.

## Learning From Failures and Setting Expectations

It is interesting that we often publish and report our successes, but very seldom our no-less valuable failures, a phenomenon sometimes referred to as the ‘file drawer problem’ (Rosenthal, 1979; Song et al., 2009). These failures often become known if they are either catastrophic or if they became failures after a period of being considered a success. Interesting examples of past failures and lessons learned come to mind. For instance, the 2008 financial crisis taught us that domain knowledge is important when applying sophisticated data-driven algorithms to complex systems. Other examples can be found in robotics (Sheh and Monteath, 2017). Closer to home, the mental health translational pipeline is hindered by our inability as a field to produce animal models of polygenic diseases that accurately reflect any human psychopathological condition (Monteggia et al., 2018; Arguello et al., 2019; Bale et al., 2019). Or vice versa, by our inability to back translate human pathophysiological findings into animal models to gain more mechanistic insights. Significant obstacles need to be overcome to understand the role of the brain in behavior, to understand disease mechanisms and to obtain sets of biomarkers capable of characterizing a mental disease state and monitor the progress of its treatment.

On the computational front, early attempts using ANNs were successfully used to provide a data-driven way to map symptoms to diagnoses of depression (Nair et al., 1999), and in a second example to predict the effect of adinazolam SR in panic disorder with agoraphobia (Reid et al., 1996). While both studies produced interesting results, neither provided any mechanistic insights into depression or panic disorder (Cohen and Servan-Schreiber, 1992). Toward this goal, we might next look to combinations of biophysically informed models with traditional deep learning methods such as CNNs. For a variety of reasons, however, for-profit companies (the most important designers and users of ML) might not want or need to create interpretable models, so the bulk of the effort may need to come from academia or public-private partnerships.

XAI might be easier to deploy in applications such as computer vision where sensory constructive hierarchies are more clearly defined and key features for classification can be found. In radiology (medical imaging), explainability is gaining interest, including in systems that learn from expert’s notes (Laserson et al., 2018). Perhaps, our desire to achieve a comprehensive theory of how brain and behavior relate to each other in more naturalistic settings might be unnecessarily ambitious, whereas well-defined and controlled experimental conditions may be as instructive of general principles.

As an initial step, new XAI projects should provide proof of concepts for new technology relevant to mental health with very narrow focus rather than immediately aiming at longer-term goals such as curing schizophrenia or major depressive disorder. They will likely focus on key behavioral components which can be improved relatively quickly. The advent of new neurotechnology (computational or not) will allow us to answer new and more interesting questions. Even with current technologies, and limited data, we can still do a lot to generate new levels of understanding by shifting current ML paradigms. Technology development is important, but alone, it will not solve the problem of intelligent and explainable neurobehavioral modulation. We also need guiding theoretical/hypothesis-driven approaches that interact with the development and implementation of data-driven technologies. There is a need for more partnership opportunities between scientific domain experts in new or established theories and ML experts. Specifically, engaging users (e.g., clinical providers, patients, researchers) is a challenging problem that highlights that cultural normalization of these approaches is at least as important as statistical normalization (e.g., collecting reference ranges for various novel metrics). Actual “Big Data” in neuropsychiatry (as in an astounding number of individuals representative of natural heterogeneity) might not be the only path forward for AI to address behavioral health issues; but, “Deep-Data” (multimodal signals collected over time within single individuals) might be more feasible now (Vu et al., 2018). One concern is that current and very successful ML tools, such as deep learning, might seem precise in classifying and predicting within a specific learned dataset, but their results are often not robust and may not generalize to other datasets. These models can indeed be easily fooled (so-called ‘adversarial attacks’) when a new source of noise is added in the system or applied to data-sets that are out of sample (Finlayson et al., 2019).

## How Do We Operationally Define the “Explainable” Part of Xai? What Are the Best Strategies for Using Successful Ai Model Constructs to Identify Concrete Causes (In Contrast to Correlations) and (Actionable) Variables?

**FIGURE 2:**
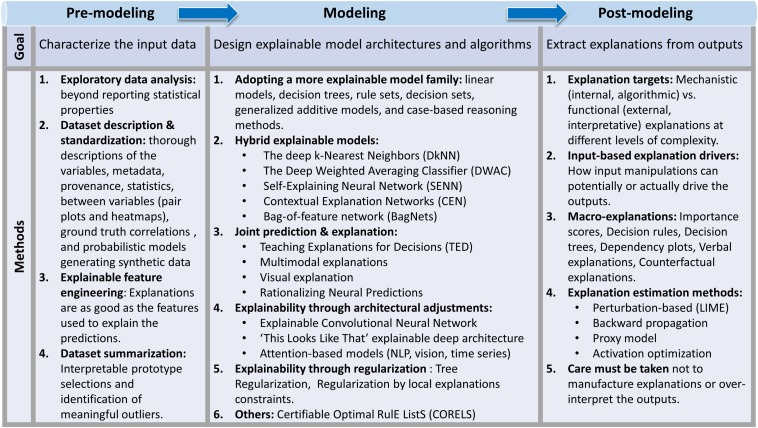
The XAI Pipeline. Explainability can be achieved at various stages of the design of a system by characterizing the input data, developing explainable architectures and algorithms, or by implementing *post hoc* algorithms. Adapted from Khaleghi (2019). Similarly, see an up-to-date public repository of implemented XAI models (https://github.com/topics/xai) and papers (https://github.com/anguyen8/XAI-papers).

There are no standard textbooks on XAI yet, but public repositories of implemented XAI models^2^ and papers^3^ are available. Similarly, attempts have been made to define explainability (Lipton, 2016; Gilpin et al., 2018; Murdoch et al., 2019) and propose practical steps that can be taken to develop an XAI system (see Figure 2, Khaleghi, 2019) and evaluate it (Doshi-Velez and Kim, 2017). The first step is to increase the information about the input datasets (Figure 2, left column). This can be achieved by preprocessing the data to extract information about its dimensionality, perhaps leading to some human-interpretable *a priori* partition (e.g., principal component analyses of input EEG channels, separation of artifacts, t-SNE (van der Maaten, 2014) or UMAP (McInnes et al., 2018) techniques). Various data visualization techniques can also be used to identify latent structures beyond those that can be obtained by straightforward statistics (Matejka and Fitzmaurice, 2017; Sarkar, 2018). Characterization of the input data can also be done by annotating and standardizing them, using for example documentation approaches such as datasheets (Gebru et al., 2018). Input data can also be embedded into a larger space in which additional dimensions are explainable features of the data (e.g., add spike burst occurrence dimension, because they may constitute privileged windows of synaptic plasticity). Such feature engineering can be done using expert knowledge in the field, or in a more principled manner using analytical approaches such as LIME (Locally Interpretable Model-Agnostic Explanations) or Contextual Explanation Networks (Al-Shedivat et al., 2018) or more general model-based techniques (Higgins et al., 2018; Murdoch et al., 2019). Finally, the explainability of the input data can be enhanced by the identification of subset of input data that are simultaneously representative of subclasses of the entire datasets and interpretable (e.g., prototypical spike waveforms sufficient for differentiating principal cells from inhibitory interneurons). Such prototypical data may serve to summarize the input dataset, and the assessment of their contributions to the model outputs can serve, at least in part, of an explanation (Bien and Tibshirani, 2011). This concept is closely related to the important topic of causality in AI (Pearl, 2009; Hernan and Robins, 2020). Equally useful to understand the data is the identification of input data that are meaningfully different from the majority of the inputs (what the data is NOT), sometime referred to as criticisms of the data (Been et al., 2016).

In addition to characterizing the data, explainability can be provided by the AI algorithm itself. Many AI models are inherently designed to potentially provide explainability, and include linear models (Ustun and Rudin, 2016), decision trees (Podgorelec et al., 2002; Geurts et al., 2009), rule sets (Jung et al., 2017), decision sets (Lakkaraju et al., 2016), Generalized additive models (Hastie and Tibshirani, 1987), and case-based reasoning systems (Lamy et al., 2019). Though potentially more explainable, these models do not guarantee explainability. High dimensionality or co-dependence of input data may make explanations difficult, if not impossible, and additional processing may be needed (Khaleghi, 2019). At least four classes of systems have been proposed that address the issue of explainability, while simultaneously attempting to maintain performance (Figure 2 middle column and Khaleghi, 2019) including Hybrid explainable models (e.g., deep weighted averaging classifier, Card et al., 2018), joint prediction-explanation models (e.g., Teaching Explanation for Decision, Hind et al., 2018), architectural explainability models (e.g., explainable convolutional networks, Zhang et al., 2018; Tang et al., 2019) and models using regularization (e.g., Tree regularization, Wu et al., 2017).

Finally, explainability can be attributed *post hoc*, by analyzing the pattern of outputs of the algorithm. Recently, Khaleghi (2019) proposed a taxonomy of post-modeling explainability approaches that we summarize next (Figure 2, right column). The first class of approaches tailors *post hoc* explanations to the target group to which these explanations are aimed: explanations that are aimed at understanding the inner workings of the algorithm (mechanistic explanations) are different from those used to inform policy makers (functional explanations and interpretations of the outputs) (Tulio Ribeiro et al., 2016; Gilpin et al., 2019). A second class of output explanation includes algorithms that rely on understanding how input manipulations can potentially or in fact drive the outputs. They include input feature section (e.g., explainable feature engineering of the inputs, above), an analysis of how specific inputs affect outputs (e.g., influence function, Koh and Liang, 2017), or an analysis of how a specific class of inputs influence the outputs (e.g., Concept activation vectors, Kim et al., 2017). A third class of algorithms are holistic in nature and includes explanatory methods that abstract or summarize the system in terms that are understandable by the user. This type of Macro-level explanations includes methods such as saliency maps (Lundberg and Lee, 2017) or Decision rules (Guidotti et al., 2018). Finally, the fourth class of *post hoc* explanatory models includes algorithms that aim at estimating (rather than providing) an explanation. These methods include generally applicable algorithms such as LIME (Tulio Ribeiro et al., 2016) or Quantitative Input influence measures (Datta et al., 2017) which uses controlled and limited perturbations of the inputs to understand how the output vary. Overall, as with the methods targeted to input data, these algorithms address the general notion of causality in AI (Pearl, 2009; Hernan and Robins, 2020).

Importantly, and perhaps similarly to many other fields, interpretation of the outputs and of the general outcomes of an AI algorithm must be checked against bias and overall exaggeration (Lipton and Steinhardt, 2018). An important issue to keep in mind when designing an XAI system is contrasting explanation, causation, and correlation. Correlation is not necessarily causal because it may be mediated by a latent, common, factor. For example, in the case that A is correlated with B because C causes A and B with some probability, C would be a partial explanation for A and B, but A and B would bear no mutual explanatory link. XAI systems should handle such differentiation, or at the very least should quantify the extent to which they occur. This issue is even more relevant in non-linear (e.g., complex recurrent) systems such as the brain. A second related outcome to such differentiation stems from the fact that the input dimensions of an XAI system are likely not independent and feature a large amount of redundancies and co-dependencies. An XAI system should be able to pair a specific explanation with a subset of the input dimensions that caused it, therefore pointing to the important dimensions to use for further study, targeted experimental manipulations, or additional focused data collection. Human-in-the-loop approaches may also be beneficial, especially in eliminating trivial correlations that may bias the system toward un-interesting solutions (Zanzotto, 2019). It is likely in fact that the process of developing explanatory power may rely on an iterative approach whereby the human would evaluate the explanation of a previous cycle, inject his/her knowledge into the XAI system, and improve the nature or accuracy of the explanation in the next cycle (Figure 1). There may be value in querying the field of Psychology of Interpretations. What makes an explanation a *good* explanation? Perhaps is it a matter of length and number of outputs explained? The more concise the explanation and the more outputs it explains, the better? Of course, explanations should be human-understandable as well (‘42’ is certainly concise and explains ‘life, the universe and everything,’ but it is hardly understandable, Adams, 1980).

Current AI can be made more explainable by using more appropriate research designs. For example, one can ask ML specific questions about brain or behavior while accounting for underlying (labeled) variables. But even the best and latest input pattern detectors, trained with multidimensional datasets will not inform us about the underlying mechanisms if we only ask how well they do at detecting the overt phenomenon. However, these detectors, when coupled to dimensionality reduction and feature extraction techniques could help identify mechanisms of action and actionable variables. Iterative feature selection and dimension reduction are methods to identify relevant features and the role played by their interactions. Another strategy could be identifying the ‘useful’ weights that contribute to the success of an AI neural-network-based algorithm and understanding what they mean in neuroscience terms and what they are doing to affect the neural circuitry. This method can address the issue of explainability as well as that of mechanism controllability. But ultimately, closed loop/perturbation experiments offer the best hope of moving beyond correlational findings. Eventually, direct and systematic mechanistic modulation of a given set of variables may be necessary to understand how the ML model reacts to each variable and each combination of variables, both in aggregate and for individual input examples. DBS systems for psychiatric disorders (e.g., OCD, MDD, Goodman and Alterman, 2012), which are first built in the clinic, will face additional challenges in the ambulatory environments. As ML takes place in these increasingly more complex environment-dependent situations, analyses of correct actions as well as errors would benefit from XAI. As an example, visual Case-Based Reasoning (CBR) – a form of analog reasoning in which the solution for a new query case is determined using a database of previous known cases with their solutions could be an effective approximation of an XAI solution that has not been employed in psychiatry (Lamy et al., 2019).

How can we determine what neural features are important to modulate behavior? The answer is likely to be different for each domain of applicability (neurostimulation or others). In general, before effective explanations can be generated, ML models should be validated (and cross-validated) on data sets not used for model-fitting, should be tested for generalizability across contexts/conditions and should incorporate strategies to avoid overfitting. The field needs to:

• Provide better analytical and statistical tools for characterizing dynamical systems within the constraints a given biological/ethological context;
• Provide models of compensation, adaptation or plasticity facilitated by exogenous modulatory inputs that might enhance (or interfere) with intended outputs and outcomes;
• Explore manifolds of parameters in unbiased ways that allow for the discovery of relevant sub-spaces where information that is biologically relevant to the organism’s existence.

## What Conceptual and Technical Advances Are Necessary for Xai to Be a Viable Component of Neurostimulation Strategies?

Perhaps the first type of advances required to make XAI a viable tool in understanding the relationships between neural circuits and behavior is an improvement in the quality and amount of the input data. The field needs more simultaneous recordings from multiple cell types within multiple brain regions comprising all putative neural circuits and a wide range of quantitative behaviors. If XAI is to find subtle and more interesting ways to understand the interaction between neural circuits and behavior, we need to find more and better way to measure them. The temporal and spatial requirements of recordings depend on the specific clinical/physiological question being asked and more, and better, data are needed for optimal explainable AI results. Temporal precision at the millisecond level and spatial resolution down to the single-neuron or microcircuit-level are likely to be necessary. Hundreds more electrodes, covering both cortical and sub-cortical areas would provide crucial information, especially in the determination of the timing and intensity of neurostimulation, in quasi-autonomous systems. Continuous data collection that enables greater sampling of key behaviors in different contexts is also likely to be able to improve the performance of such systems.

Importantly, XAI needs to be able to effectively handle multi-modal data (e.g., visual, auditory, clinical). It should provide inherently non-linear computational algorithms that will be able to combine large datasets such as those provided by modern calcium imaging techniques [>1000 of neurons recorded simultaneously (Soltanian-Zadeh et al., 2019; Stringer et al., 2019)] and voltage sensitive dye techniques (Grinvald et al., 2016; Chemla et al., 2017) with smaller but highly meaningful datasets such as those describing behavior. These improvements would result, in turn, in better ways to ‘close the loop’ and devise effective algorithms for neurostimulation. Additional advances in real-time encoding of the environment and real time classification of behavioral states would give rise to a new generation of neurofeedback systems that could be used for therapeutic purposes, greatly expanding on the current trends for adaptive neurostimulation (Provenza et al., 2019). Another challenge is to quantify behavior and neural activity at multiple levels of complexity and multiple time scales and use new statistical and analytical tools to link and compare the different levels. At each of these levels, effort should be made to differentiate externally generated influences and internally generated computations. Finally, efforts need to be made to understand the organism’s response to more naturalistic environments and stimuli. This is crucial in cases where social interactions are known to play a major role, since much of the neuroscientific data is usually collected in single subjects, or in impoverished social or cognitive environments. Finally, advances in the quality and type of data should be accompanied with advances in AI/XAI theories and ML techniques (Greenwald and Oertel, 2017).

An interesting avenue to explore is the mapping between the XAI system and the neural systems, perhaps even designing such a system from scratch, with the brain as a starting point. In the specific context of neurostimulation, better models are needed to understand how neurostimulation actually affects the neural tissue (neuron, glial cells, synapse). A challenge will be for XAI to provide explanations for phenomena known to have none (consensually at least).

In general, XAI models should be scalable to bridge animal research and human clinical applications and be sufficiently computationally efficient to allow for implementations on actual small-scale devices that can be used clinically. Improvements in sustainable high-density recording devices for humans, mirroring those already available in animals, is desirable.

Moving forward, what types of initial steps can be taken to link XAI to the field of closed-looped neurostimulation? One can certainly imagine simply applying existing or novel XAI techniques to a known neurostimulation paradigm to provide explanatory power to close-loop neurobehavioral modulation (e.g., counter-factual probes). Other avenues may involve active modulations of complex neural circuits pertaining to mental disorders. Such manipulations may involve electrical or magnetic stimulations, optogenetics, genome editing or pharmacological compounds and may include dynamic automatic adjustments of closed-loop parameters as the neural substrate adapts to the manipulations.

## Beyond Mental Health, What Other Diseases Could Benefit From an Xai Solution?

There is potentially a variety of medical conditions that could be informed by XAI. Biomarkers, broadly defined as biological measurements that give the ability to predict, detect, and diagnose, can be key targets of XAI approaches. Specific clinical domains such as epilepsy have already benefited from relatively simple closed loop paradigms (so called ‘responsive neurostimulation’ techniques). Other domains such as cardiovascular illness, infectious disease, and epidemiology could also significantly benefit. Mental health conditions, and the RDoC are of particular interest, because they focus on understanding the nature of mental illness in terms of varying degrees of dysfunctions in general psychological/biological systems (Kozak and Cuthbert, 2016; Sanislow et al., 2019). Indeed, in the absence of a very large number of behaviors and comprehensive cell-type specific measurements, we can reasonably start with chunks of behavior as conceptualized and cataloged by RDoC which does allow for a systematic approach for XAI models and experiments. Research needs to be both rigorous and pragmatic about whether supervised or unsupervised XAI models are used but should remain realistic about the level of spatial and temporal resolution possible with the current generations of human recording and stimulating devices. The ability to utilize XAI results in a closed-loop fashion can make major contributions to epilepsy treatment, for example, by preventing seizure activity using XAI-based predictions to activate an implanted neurostimulator in real time. XAI can improve the efficacy of brain stimulation devices by allowing an in-depth dissection of the networks and nodes underlying brain-based disorders, and by providing an avenue of translation between recording and stimulation architectures.

Another area most amenable to XAI approaches includes computer vision approaches to radiological imaging interpretation. This area has already seen important progress, including FDA approved tools, see for example (Topol, 2019) for a recent review, which includes important caveats. XAI can further contribute to the difficult problem of data fusion of heterogeneous multimodal measurements including, for example, simultaneously sampled imaging, neurophysiological and behavioral data.

There is a strong desire to build what is already known into models and to start from simpler scenarios. Prior data could be used to design the model, provide initial constraints, and provide error refinement. Insights from biology, such as reafference/corollary discharge and statistical models of neural firing are certainly a source of useful design information. Seeking insights from development (e.g., differences in learning during childhood vs. adulthood) can also be used as a means to inform the XAI system. Whatever the prior information, its origin should be quantitatively and objectively measured and be based on continuous behavior and neural data. Moreover, it must be kept in mind that not all cognitive measures include relevant information and care should be taken when selecting them for processing to avoid potential issues affecting interpretability Also, summary or composite measures such as those related to emotional state or context could help differentiate normal from abnormal responses and should be considered as well. Finally, the ability to handle and benefit from incomplete or uncertain data may be a major contribution of XAI approaches.

In general, XAI has the potential to contribute to the integration of data-driven and theory driven models (e.g., integrating Deep Learning models with biophysically informed models), to label existing model features with semantic information that is understandable by users, to allow ML algorithms to unbiasedly discover the governing principles of a complex dynamical system by analyzing covarying multimodal data or to estimate the influence of a given feature on a model prediction by leveraging causal statistical methods.

## Concluding Remarks

One key proposed approach to stimulate the field is the establishment of competitions on existing (curated) datasets, an approach that has been very successful in other disciplines (e.g., computer vision and ML). Other disciplines have shown multiple benefits of this type of activity, including the possibility to compare and merge results and outcomes from multiple teams, the opportunity to show and evaluate progress, and the motivation experienced by atypical contributors that enjoy such competition and enter a field. Areas such as closed loop-neurostimulation provide multiple challenges, and openly sharing data via competitions can bring together multiple disciplines addressing problems ranging from signal synchronization to optimal outcome analysis and stimulation settings. Initial attempts in this direction in neuroscience recently started, and include a number of EEG competitions^4^ and spike inference for calcium imaging (Berens et al., 2018).

It is important to note the need to harmonize different types of data and the necessity of longitudinal multimodal data. There is a large amount of existing data that can be tapped for secondary analyses, including the aforementioned competitions (e.g., the ENIGMA project)^5^. Aggregation needs to happen across scales, time (longitudinal), and individuals. The potential value of explainability in this challenge is clear; it is expected that the more explainable the data and analyses are, the easier it will be to combine disparate sources.

Following the trend of using and sharing existing data, there is a need to study “hybrid models” which use AI approaches to fit a biologically driven model – does AI converge on the same solution as expected? A recent example has been published (Banino et al., 2018; Cueva and Wei, 2018). Neurostimulation is a good sandbox where ML and biology are starting to interact (Kim et al., 2019; Shamir et al., 2019), and for which the need of explainability of biomarkers and interventions is critical.

It is important for researchers to be aware of the pitfalls inherent to the translation of results and models from animals to humans and the need to collect data with multiple tools and open technologies, staying away as much as possible from proprietary tools. This “closed” practice can lead to fitting to correlated noise in datasets/variation that is not biologically/clinically meaningful and to limit reproducibility and validation. The above mentioned openly shared and combined datasets is an important contribution to the development of better XAI.

Unsurprisingly, explainable AI in neuroscience and neurostimulation suffer from the ‘curse of dimensionality’ (data of very high dimensions), and partially driven by this challenge, show the need to consider simpler models, including variable selection. While this is an example of a technical/computational problem, clinical failures from the past need to be addressed as well, in particular the need to avoid the expectation that neurostimulation must have immediate effects (as in DBS for PD), but rather has complex and mixed acute and chronic effects, possibly involving long term synaptic plasticity. Using a single outcome measure, as was often done in the past, can lead to incorrect conclusions about models and interventions; there is a need to incorporate measures at multiple time scales, to use derivative-based metrics, to measure rate of change and to build characterization of normative data so as to measure deviations from it. It is interesting to note that these issues, here mentioned as failures from the past, connect to the above identified need to integrate data from multiple sources, time resolutions, and spatial scales, which is a recurring concern and for which explainability can be of significant help.

In addition, explainability may be valuable for building trust in the algorithms, for understanding risk and side effects, for aiding in the identification of therapeutic targets, for understanding the evolution or progression of disease and response to treatments, for understanding and supporting decisions, for closed-loop control, and for the design of the “safety parameter box” – FDA’s bound on therapies. Although explainability may lead to improved trustworthiness, transparency and fairness, these are distinct but related concepts. The predisposition of scientists and healthcare professionals to accept the validity and reliability of ML results, given changes in the input or in the algorithmic parameters, without necessarily knowing how the results were derived has to do with trustworthiness. Trust relies on five key factors: the data, the system, the workflow, the outputs, and the ability to communicate the results of the algorithm clearly. Users need to be able to probabilistically determine when some results might be incorrect and ensure that results are interpreted correctly without needing to know the inner workings of the algorithm. Transparency and Fairness relate to the right to know and to understand the aspects of a dataset/input that could influence outputs (e.g., clinical decision support from AI algorithms or neurostimulation protocols). Transparency and fairness should lead to a reduction of bias perpetuation that can be produced by humans (e.g., tracking and education regarding biases in language), by AI algorithms (e.g., developing AI approaches able to identify bias in results), by better data collection (e.g., utilize more representative data sets).

It is of course critical to keep in mind that explainability can be beneficial but is not mandatory (e.g., detecting amyloid plaques in Alzheimer’s Disease imaging data). In other words, non-explainable (or non-explainable yet) predictions can still have value as biomarkers. Importantly, explainability might be different for different audiences (Tomsett et al., 2018; Gilpin et al., 2019). For example, what needs to be explainable for the FDA might be different than for scientists or even patients (Murdoch et al., 2019), and these discrepancies raise regulatory issues related to the ‘right to explanation’ (Goodman and Flaxman, 2016). Finally, the incorporation of explainable ML in clinical trials, for example, to optimize neurostimulation parameters in a patient specific fashion instead of the common use of fixed protocols, can be a novel direction of research. This brings us to the important current area of AI in drug design, a very active topic of research in the academic and even more in the industrial community (Simm et al., 2018).

In sum, XAI applied to the domain of closed-loop neurostimulation may yield important new insights both at the fundamental research level and at the clinical therapeutic level and is ideally positioned to generate a new set of translational approaches capable of using increasingly larger multi-modal datasets to discover basic principles about normal and abnormal brain functions.

## Ethics Statement

Written informed consent was obtained from the individual(s) for the publication of any potentially identifiable images or data included in this article.

## Author Contributions

All authors contributed to manuscript conception, design, writing, literature review, and revision. All authors read and approved the submitted version.

## Conflict of Interest

The authors declare that the research was conducted in the absence of any commercial or financial relationships that could be construed as a potential conflict of interest.

## Footnotes

1 https://grants.nih.gov/grants/guide/pa-files/PAR-19-344.html

2 https://github.com/topics/xai

3 https://github.com/anguyen8/XAI-papers

4 https://www.kaggle.com

5 http://enigma.ini.usc.edu/

## Abbreviations

AI: Artificial Intelligence
ADHD: Attention Deficit and Hyperactivity Disorder
ANN: artificial neural networks
BMI: brain machine interface
CNN: convolutional neural networks
DBS: deep brain stimulation
ECoG: electro corticogram
EEG: electro encephalogram
FDA: Food and Drug Administration
fMRI: functional magnetic resonance imaging
MDD: major depression disorder
ML: machine learning
MVPA: multi variate pattern analysis
OCD: obsessive compulsive disorder
PD: Parkinson’s disease
RDoC: Research Domain Criteria
SR: slow release
SVM: support vector machine
t-SNE: t-Stochastic Neighbor Embedding
UMAP: Uniform Manifold Approximation and Projection
XAI: Explainable Artificial Intelligence.
